# Bubble-propelled micromotors for ammonia generation†

**DOI:** 10.1039/d3nr03804a

**Published:** 2023-09-18

**Authors:** Rebeca Ferrer Campos, Harshith Bachimanchi, Giovanni Volpe, Katherine Villa

**Affiliations:** a Institute of Chemical Research of Catalonia (ICIQ) Av. Països Catalans 16 Tarragona E-43007 Spain kvilla@iciq.es; b Department of Physics, University of Gothenburg Origovägen 6B Gothenburg 41296 Sweden giovanni.volpe@physics.gu.se

## Abstract

Micromotors have emerged as promising tools for environmental remediation, thanks to their ability to autonomously navigate and perform specific tasks at the microscale. In this study, we present the development of MnO_2_ tubular micromotors modified with laccase for enhanced oxidation of organic pollutants by providing an additional oxidative catalytic pathway for pollutant removal. These modified micromotors exhibit efficient ammonia generation through the catalytic decomposition of urea, suggesting their potential application in the field of green energy generation. Compared to bare micromotors, the MnO_2_ micromotors modified with laccase exhibit a 20% increase in rhodamine B degradation. Moreover, the generation of ammonia increased from 2 to 31 ppm in only 15 min, evidencing their high catalytic activity. To enable precise tracking of the micromotors and measurement of their speed, a deep-learning-based tracking system was developed. Overall, this work expands the potential applicability of bio-catalytic tubular micromotors in the energy field.

## Abstract 2

Here, we introduce self-propelled biocatalytic micromotors for simultaneous organic pollutant removal and green energy generation. The study demonstrates remarkable results, showcasing the potential to generate ammonia from wastewater in short time.

## Graphical abstract

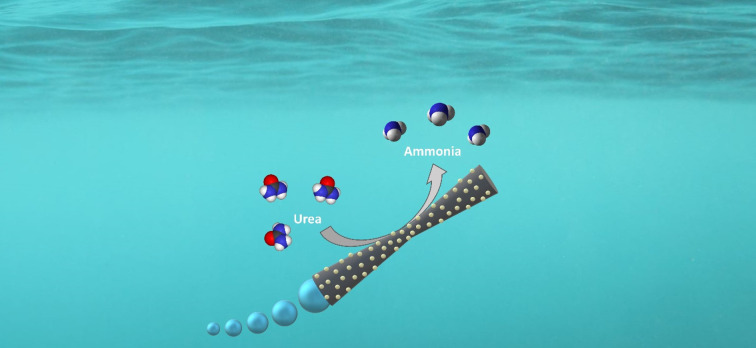

## Introduction

Anthropogenic and industrial activities generate a great variety of contaminants that are released into water bodies. Since these pollutants cannot be removed by current wastewater systems, they remain in the environment resulting in environmental challenges that negatively affect the different water sources and our health. Many studies have been carried out in the field of environmental remediation for the removal of organic pollutants from contaminated water, including pharmaceutical, agriculture, and dye waste sources.^1–6^

Among these emerging contaminants, urea has been gaining prominence because it is a common pollutant from residential activities, being the main component of urine, and from different industrial processes. Therefore, the accumulation of urea in the wastewater of big cities is becoming a great problem because its biodegradation is not enough to avoid related environmental risks, like eutrophication in coastal waters.^7–9^ To overcome this issue, an effective, affordable and environmentally friendly approach is needed. A plausible solution to overcome urea contamination deals with its conversion into ammonia. Such chemical transformation entails two main advantages in one single process: removal of a pollutant and generation of a renewable carbon-free fuel.

Ammonia has been gaining importance in the fields of power generation and storage as a carbon-free green fuel.^10–12^ It has a high energy density and it can be catalytically decomposed for the production of CO_*x*_-free hydrogen or used directly as liquid fuel for power or electricity generation, even in many currently available power-generation facilities. Moreover, the infrastructure for liquified ammonia transportation and storage are already in place and well established, which is a major advantage towards its use.^13,14^ A recent study showed the feasibility of converting urea into ammonia by (photo-)catalytic reactions using TiO_2_.^2^ However, it requires long reaction times and light as an extra energy input. Therefore, more efficient systems are still needed.

Self-propelled micromotors are well-known to enhance mass transfer processes, due to their active motion.^15^ As a result, they have been widely explored for the removal of different types of pollutants, such as dyes,^16,17^ heavy metals,^18,19^ and oil spills.^20^ Among the different types of catalytic micromotors,^21^ MnO_2_ has shown many advantages compared to other materials for the synthesis of micromotors. It provides a high specific surface area, has good chemical stability and is a low-cost material. Moreover, its capacity to oxidize organic compounds and self-propel in the presence of H_2_O_2_ has promoted the use of MnO_2_-based micromotors in the environmental field as an alternative to Pt-based micromachines.^22–26^ On the other hand, the immobilization of enzymes on the surface of micro/nanomotors have shown to enhance the removal yields of several organic pollutants and oil-based compounds.^27^ For instance, laccase is a multi-copper oxidase that has the ability to oxidize various substrates. To increase reusability and recovery, different approaches involving the immobilization of laccase on the surface of various materials and micromotors have been reported over the last years.^28–33^ Moreover, lipase-based nanomotors have shown promising degradation rates of triglycerides,^34,35^ expanding their applicability not only in biomedicine^36^ but also for the removal of oil spills.^37^

**Fig. 1:**
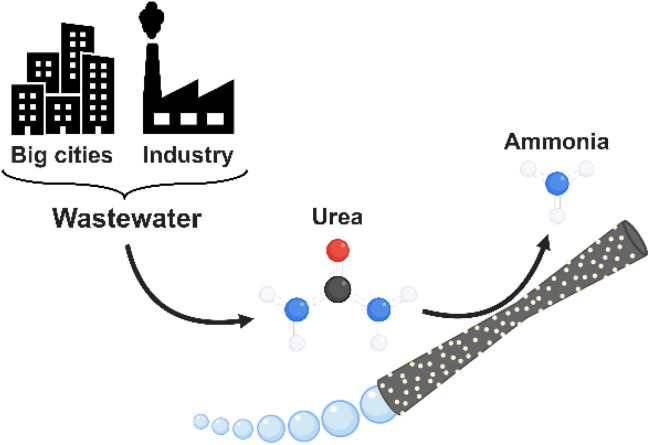
Ammonia generation and pollutant removal by MnO_2_-based tubular micromotors.

In this work, we introduce hybrid tubular micromotors, based on a MnO_2_ catalytic component, and decorated with laccase as the bio-catalytic counterpart (MnO_2_/Lac micromotors) for the generation of ammonia from urea, a widely available contaminant in wastewater (Fig. 1). Therefore, this work explores a novel application of self-propelled micromotors based on bio-catalytic components for environmental remediation as well as for green energy production.

## Experimental

### Fabrication of MnO_2_/Lac micromotors

**Fig. 2:**
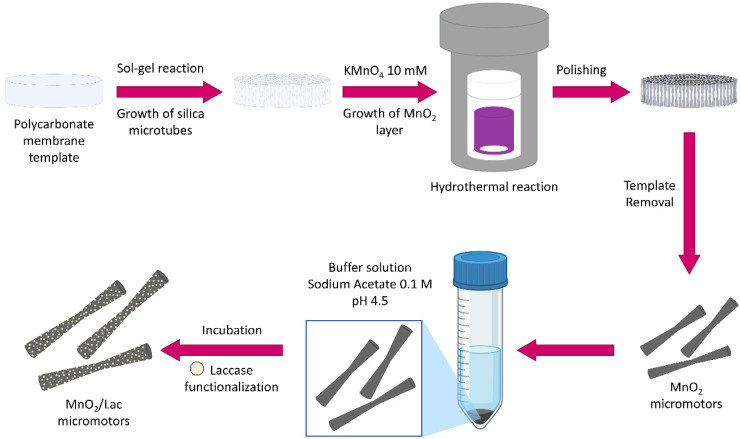
Fabrication of MnO_2_-based micromotors by wet chemical methods followed by laccase immobilization.

For the synthesis of MnO_2_/Lac micromotors (Fig. 2), micro-silica tubes (MSTs) were firstly grown inside a polycarbonate membrane using a mixture of cetyltrimethylammonium bromide (CTAB), triethanolamine (TEOA), 3-aminopropyl triethoxysilane (APTES), and tetraethyl orthosilicate (TEOS), as described in a previous study.^38^ Then, the membrane with the grown MSTs was introduced into a Teflon flask with 30 mL of 10 mM KMnO_4_ to obtain the MnO_2_ on their surface. The flask was placed inside a stainless-steel autoclave and heated at 160 °C for 9 h.^26^ Once the polycarbonate membrane was dissolved, the surface of the as-synthesized MnO_2_ micromotors was functionalized with laccase from Trametes versicolor by adding them to 10 mL of 0.1 M sodium acetate/acetic acid buffer solution at pH 4.5 containing 2 mg of the enzyme. The solution was incubated for 15 minutes at 25 °C and 200 rpm and then centrifuged for 2 minutes at 6000 rpm. The functionalized micromotors (MnO_2_/Lac micromotors) were finally washed with water.

### Characterization of MnO_2_-Lac micromotors

The chemical composition and morphology of the micromotors were examined by a high-resolution field emission scanning electron microscope with a focused Ga ion beam (FESEM-FIB, Scios 2 by FEI Company), and the chemical analysis was performed using a FESEM Inspect F50 with an EDS detector (EDAX). A transmission electron microscope of atomic resolution (FETEM) (JEOL F200) with an EDX Centurio detector (silicon drift) was used to perform elemental analysis. The powder X-ray diffraction data was obtained using a Bruker AXS D8-Discover diffractometer (40 kV and 40 mA).

The motion speeds of the micromotors were estimated from the videos recorded using a confocal microscope (Nikon TE 2000E) coupled to a halogen lamp and a Hamamatsu camera. The velocities of the micromotors with manganese oxide were estimated with hydrogen peroxide concentrations of 2%, 5% and 7% (w/w), while the velocities of the micromotors with laccase were estimated only with a concentration of 2% (w/w) H_2_O_2_. The results are from an average of 20 different micromotors for each concentration.

### Micromotor tracking

**Fig. 3:**
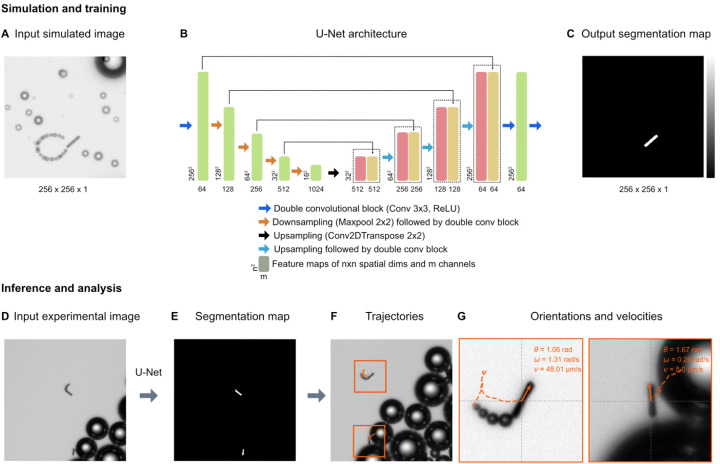
Deep learning-based micromotor tracking. Simulation and training: (A) Example of a simulated micromotor image generated by DeepTrack 2.1.^33,34^ Each image (256 px × 256 px) contains one or more micromotors with aspect ratios similar to those used in the experiments. Bubbles of different sizes (smaller bubbles trailing behind the rods, and larger bubbles in the background) are added to make the images look realistic and thereby closer to the experimental images (see the experimental images in (D) and (G)). For each input image (A), a corresponding output image with a segmentation map (C) is also generated to separate the micromotors from the background and the bubbles. (B) U-Net architecture: A U-Net is employed to transform the input simulated image into the segmentation map. The architectural details and information on each block are indicated in the legend. Inference and analysis: the U-Net model trained on simulated images is then applied on experimental images (D) generating the segmentation maps of the micromotors (E). The positions and orientations of the micromotors are obtained from the segmentation maps. (F) Trajectories: by extracting the positions of all the frames in a video, the trajectories are measured. (G) Each trajectory is further processed to calculate the linear velocities (*v*) and angular velocities (*ω*). Individual micromotor trajectories are represented in the orange boxes.

For the tracking of the micromotors, we used a deep-learning-based analysis approach. Fig. 3 shows an overview of the analysis pipeline to estimate the micromotor trajectories along with their 2-dimensional translational and angular velocities. To detect the micromotors from the videos, each frame in the video (Fig. 3A) is analysed by a U-Net (Fig. 3B). A U-Net is a fully convolutional neural network that was originally developed for the semantic segmentation of biomedical images.^39^ The output of the U-Net is a heatmap containing the segmentation of the micromotors (Fig. 3C). The U-Net is trained on simulated input–output image pairs (see Fig. 3A and C and section ‘Simulation and U-Net training’) that are generated by utilizing the optics engine and microscopy pipeline of the Python software package DeepTrack 2.1.^40,41^ Unlike traditional methods, training a neural network on custom simulated dataset helps to segment the objects of interest (the micromotors) from the background (the bubbles) with an improved detection accuracy. Upon training, the U-Net is used to process the experimental data. Fig. 3D shows an example experimental image. The output of the U-Net as shown in Fig. 3E depicts the segmentations of the micromotors while disregarding the background illumination and the microbubbles. Notice two different kinds of bubbles in the image (larger bubbles in Fig. 3D and trailing bubbles at the end of the micromotors, indicating the propulsion in Fig. 3G) that are discarded in the segmentation map (Fig. 3E) generated by the U-Net. The segmentation map is then used to obtain the 2d positions of the micromotors and their orientations. The process is repeated for all frames in the video through batch processing (see section ‘Simulation and U-Net training’) to obtain the positions and orientations of micromotors in all frames. To obtain the trajectories (See Fig. 3F), the positions of the micromotors in subsequent frames are linked through a custom Python implementation of the Hungarian algorithm. The trajectories are further analysed by considering the frame rate (7 fps), and effective pixel size of the microscope (1 px = 1.53 μm) to obtain the instantaneous linear and angular velocities of the micromotors (Fig. 3G). Fig. 3G shows the individual trajectories of micromotors that are detected in Fig. 3D. The inset shows the instantaneous orientations (*θ*), angular velocity (*ω*) and translational velocity (*v*) of each micromotor.

### Simulation and U-Net training

**Fig. 4:**
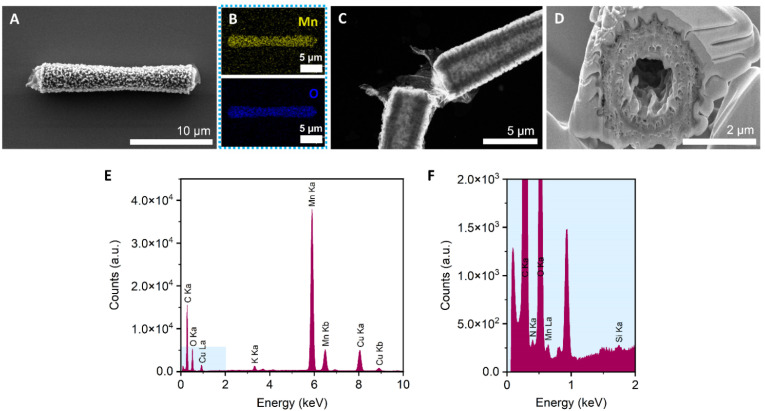
Characterization of MnO_2_/Lac micromotors. (A) High-resolution FESEM image of a MnO_2_ micromotor after the hydrothermal reaction. (B) EDS-mapping of A, showing the Mn (yellow) and O (blue). (C) FETEM image of a MnO_2_ micromotor after laccase immobilization. (D) Cross-section of a MnO_2_ micromotor. (E) Elemental analysis spectrum of a MnO_2_/Lac micromotor. (F) Magnification of blue square in figure (E) from 0 to 2 keV.

The U-Net is trained on simulated input–output pairs of micromotor images (Fig. 3A) and the corresponding segmentation maps (Fig. 3C). Each simulated image of size 256 px × 256 px contains micromotors of dimensions (15 μm × 2 μm) similar to the experimental micromotors (Fig. 4A). Microbubbles of different sizes are simulated to make the images realistic and close the experimental images. In order to train the network to disregard the trailing bubbles in the segmentation maps of micromotors (notice the bubbles behind the micromotors in Fig. 3G) considering their close proximity to the rods, microbubbles of similar size are simulated at the distal ends of the micromotors’ centroids (Fig. 3A). The orientations and paths of trails are fine tuned to mimic the micromotor motion. All the simulations are performed by creating custom scatterers (for example, rod-like scatterers, spheres, and disks) in the DeepTrack 2.1 software package and are processed through the software's numerical microscope pipeline to generate the images.^40,41^ The positions and orientations of micromotors and positions of background bubbles are randomly sampled from a normal distribution. The positions of the trailing bubbles are sampled based on the position of the micromotors and a randomly sampled orientation angle for the trail path. To make the network focus on the micromotors, the output segmentation maps contain only the outlines of the micromotors (Fig. 3C).

The U-Net architecture used in the analysis is shown in Fig. 3B. In the downsampling part, the input image is downsampled by a series of convolutional blocks (indicated by blue arrows) followed by max pooling operation (indicated by orange arrows). Each convolutional block contains a fixed number of convolutional layers followed by a ReLU activation. We use a sequence of five convolutional blocks containing 64, 128, 256, 512, and 1024 convolutional filters, respectively. The outputs of each convolutional block, called as feature maps, are indicated in green blocks with the shape of the outputs indicated at bottom. In the upsampling part, we use a series of four upsampling blocks (indicated by black and cyan arrows) to convert the feature maps to the segmentation maps. Each upsampling block contains a convolutional transpose operation followed by convolutional blocks with 1024, 512, 256, and 128 convolutional filters, respectively. At each step in the upsampling part, the feature maps obtained from the downsampling part (indicated by green) are appended to the upsampled features. The outputs of the upsampling blocks are indicated in red, and the appended feature maps are indicated in yellow. In the end, a series of two convolutional blocks of dimensions 64 and 1, are applied to transform the feature maps back to the size of the original image (256 px × 256 px). A sigmoid activation is applied to the final output. The U-Net is trained using the AMSgrad optimization algorithm^42^ at learning rate of 0.0001. The network is trained on 1024 input–output image pairs in mini-batches of 64 images for 30 epochs, with a binary cross entropy loss function. The training process including the data generating process takes less than 30 minutes on a server equipped with Nvidia A 100 graphics processor unit and AMD EPYC 7302 16-core CPU.

### Degradation of rhodamine B

The MnO_2_ and MnO_2_/Lac micromotors were added to a solution containing 10 ppm of rhodamine B (RB) and UV-vis measurements were taken with a UV–vis spectrophotometer (Shimadzu Corporation UV-1800PC spectrophotometer instrument). The measurements were taken after 3 h of reaction. Several blanks were prepared and analysed, including a 10 ppm rhodamine B solution, a mixture of rhodamine B (10 ppm) and H_2_O_2_ 2% (w/w), and a 2 mL mixture of rhodamine B (10 ppm) with 2 mg of laccase.

### Ammonia generation

The conversion of urea into ammonia was quantified by ion chromatography after 15 min of reaction with an HPLC 930 Compact IC Flex series (from Metrohm and controlled by MagIC software). The mobile phase used was a mixture of 3 mM HNO_3_ and 1 mM oxalic acid, with a flow of 0.9 mL min^−1^, and the column used was a Metrosep C 6-150/4.0. For the quantification of nitrite and nitrate ions, the mobile phase used was 3.2 mM Na_2_CO_3_ and 1 mM NaHCO_3_, with a flow of 0.7 mL min^−1^, and the column used was a Metrosep A Supp 5-250/4.0 + A Supp5-150/4.0. The blank samples were a mixture of urea (1 mg mL^−1^) with H_2_O_2_ at 2% (w/w).

The amount of urea in solution after the reaction was determined using a UV–vis spectrophotometer (Shimadzu Corporation UV-1800PC spectrophotometer instrument) following a method previously described in the literature.^43^ The calibration curve and all the stock solutions were prepared as stated in the literature (HCl 2 M, methyl orange 6 × 10^−4^ M, KBrO_3_ 10^−3^ M) except for the urea, the concentration of which was of 1 mg mL^−1^ in all the standards, blanks and samples. All the blanks were prepared by adding the reagents to the urea solution in the following order: 1 mL HCl 2 M, 1 mL methyl orange 6 × 10^−4^ M, 1 mL KBrO_3_ 10^−3^ M. In the case of the samples, the measurements were done after the urea to ammonia conversion reaction. After the 15 min reaction, the general procedure was to filter the solution to separate it from the micromotors and stop the reaction, and the reagents for the urea determination were added to the solution to start the measurements.

### Evaluation of radical generation

The hydroxyl radicals (˙OH) generated by the micromotors were studied by fluorescence using terephthalic acid (THA) as a probe molecule, as described in a previous study.^44^ The fluorescence measurements were carried out on a Fluorolog Horiba Jobin Yvon spectrofluorometer equipped with a photomultiplier detector, double monochromator and xenon light source. Additionally, the micromotors were characterized by electron paramagnetic resonance spectroscopy (EPR) with an EMX micro spectrometer with an X-band bridge of 9.1–9.9 GHz. DMPO was used as a spin trap as described in the literature.^45,46^ A 100 mM solution of DMPO was used for the reaction, together with H_2_O_2_ (2% (w/w)) and the MnO_2_-Lac micromotors. Then, the EPR measurement was performed. Additionally, two blanks were measured, including a mixture of H_2_O_2_ (2% (w/w)) with the DMPO, and a mixture of MnO_2_ micromotors, H_2_O_2_ (2% (w/w)) and the DMPO.

## Results and discussion

### Characterization of MnO_2_-based micromotors

MnO_2_ tubular micromotors were prepared by a combination of sol–gel procedures and hydrothermal reactions, as described in the Experimental section.^26,38^ The loading of MnO_2_ into the silica microtubes was optimized in our previous work,^26^ by changing the concentration of KMnO_4_ from 10 to 30 mM. As a result, 10 mM was chosen as the optimal concentration of the MnO_2_ precursor for obtaining micromotors with high motion speeds. The surface of the micromotors was then functionalized with laccase by following an adsorption procedure previously described in the literature with slight modifications.^47^ The morphological, structural and chemical characterization of the micromotors is shown in Fig. 4. To confirm the tubular structure and the optimal loading of MnO_2_ inside the tubes, FESEM images were taken after each step. The as-synthesized silica microtubes consisted of a biconical structure and exhibited a rough surface with a diameter of 2 μm and 15 μm length (Fig. S1†). Fig. 4A shows the morphology of the MnO_2_ micromotors after the hydrothermal reaction. Unlike previous works,^26^ the manganese oxide seems to grow not only inside the tubes but also on the outer surface of the silica tubes. Fig. 4B shows the EDS-mapping of the MnO_2_ micromotors, evidencing the presence of Mn and O elements. As shown in Fig. 3C, the tubular MnO_2_ micromotors present a hollow structure, as corroborated by FETEM. To get insights on the inner surface of the micromotors, a cross-section of the tubes was obtained by beam cutting (FESEM-FIB, using a Ga ion beam (Fig. 3D)). The tubes exhibit a rough inner surface and a circle of a different material inside the porous MnO_2_, attributed to the silica microtube used as a scaffold.

As can be seen from Fig. S2,† no differences on the morphological structure of MnO_2_ micromotors before and after surface modification with laccase were observed. Additionally, to the FETEM images, elemental analysis spectra were obtained with the EDX detector. As shown in Fig. 4E, the silica and nitrogen elements were detected in very small amounts, confirming the presence of Si from the tubes and the presence of the enzyme on the modified micromotors.

The crystallinity of the MnO_2_ micromotors was analyzed by X-ray diffraction. As can be seen from Fig. S3,† the micromotors present an amorphous crystalline structure, and the diffraction peaks correspond to the monoclinic crystalline phase of K_0.5_Mn_2_O_4_·1.5H_2_O (JCPDS card number 00-042-1317). The stoichiometry confirms that the sample contains manganese(iv) oxide but is not pure since it contains potassium coming from the aqueous solution of KMnO_4_ used for the hydrothermal reaction, which also explains the presence of water.

### Motion characterization

**Fig. 5:**
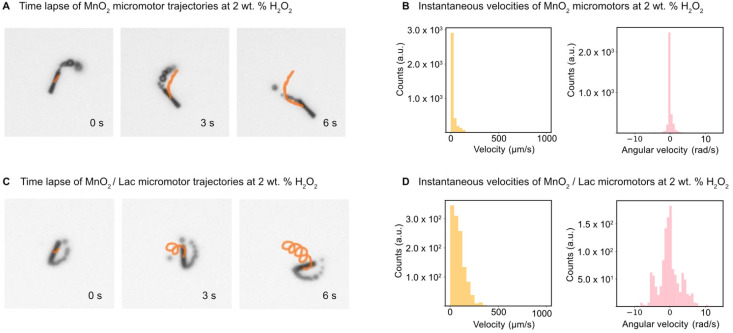
Motion characterization of MnO_2_ and MnO_2_/Lac micromotors at 2 wt% H_2_O_2_. (A) Time-lapse of MnO_2_ micromotors trajectories. (B) Velocities and angular velocities for MnO_2_ micromotors. (C) Time-lapse of MnO_2_/Lac micromotors trajectories. (D) Velocities and angular velocities for MnO_2_/Lac micromotors.

The motion of the MnO_2_ micromotors and MnO_2_/Lac micromotors was evaluated in the presence of different concentrations of fuel (hydrogen peroxide), as described in the experimental part. First, we examined the minimum required concentration of H_2_O_2_ for the micromotors to move, which was found to be 2% (w/w). Then, the velocities of MnO_2_ micromotors were characterized at different concentrations of H_2_O_2_, including 2%, 5% and 7% (w/w) by the approach described above (see sections ‘Micromotor tracking’ and ‘Simulation and U-Net training’, and Video S1†). As can be seen from Fig. S4,† there is an increase in the speed of the micromotors by increasing the amount of fuel, reaching a maximum value of 750 μm s^−1^ at a concentration of 7% (w/w) H_2_O_2_. The micromotors exhibit circular and linear motion behaviours (Fig. 5A and C). To compare the effect of the laccase loading on the surface of the micromotors, the speed of MnO_2_/Lac micromotors was also analyzed at 2% H_2_O_2_. The histograms in Fig. 5B, D and S4B, S4D,† depict the instantaneous velocities and angular velocities of the micromotors detected from 20 different videos recorded for each concentration. As can be seen from Fig. 5 and Video S2,† the presence of laccase has a positive effect on the motion behaviours, since they showed higher motion speeds in comparison with bare MnO_2_ micromotors. This enhancement might be attributed to the capacity of laccase to decompose H_2_O_2_ by a Fenton-like mechanism, due to the presence of copper atoms in their structure.^48–50^ Additionally, upon modification, the micromotors move mostly by circular motion patterns, leading to higher angular velocities than the non-functionalized ones.

### (Bio-)catalytic performance of MnO_2_-based micromotors

**Fig. 6:**
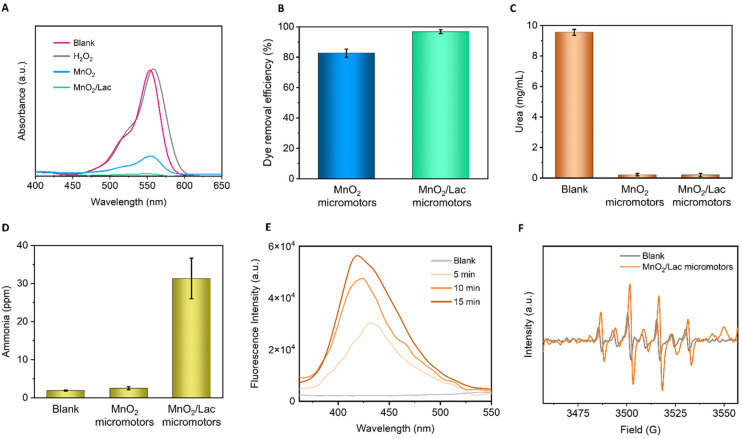
Catalytic performance of MnO_2_-based micromotors. (A) UV-vis spectra of RB degradation experiments after 3 h of reaction and dye removal efficiencies (inset). (B) Dye removal efficiency of the MnO_2_ and MnO_2_/Lac micromotors after 3 h of reaction with RB. (C) Amount of urea left in solution after 15 min of reaction. (D) Ammonia generated in ppm after 15 min of reaction. (E) Fluorescence spectra of the terephthalate monohydroxylate isomer along the 15 min of reaction time. (F) EPR spectra of the generated ˙OH radicals. Error bars correspond to the standard deviation of the mean (*n* = 3).

The bio-catalytic activity of the as-synthesized micromotors was first evaluated in the presence of Rhodamine B (RB), which is a common pollutant from textile industries. The reaction was left for 3 h before measuring to ensure that the micromotors were moving in the solution and not through the foam generated at the start of the reaction, due to the rapid H_2_O_2_ decomposition. As shown in Fig. 6A, the blank composed of only H_2_O_2_ did not degrade RB. Additionally, the combination of RB with free laccase only showed a negligible decrease in RB concentration (Fig. S5†). However, the addition of MnO_2_/Lac micromotors led to a total degradation of RB, exhibiting superior performance in comparison with bare MnO_2_ micromotors, as shown in Fig. 6B.

Once we confirmed the high oxidative capabilities of our micromotors, we further evaluated their performance towards ammonia generation from urea, as a waste pollutant. For this purpose, we combined the micromotors with urea (1 mg mL^−1^) in the presence of the H_2_O_2_. A blank experiment in the absence of micromotors was also carried out. The reaction was performed for 15 min, and then an aliquot was taken and analyzed by ion chromatography and UV-vis spectroscopy. As can be seen from Fig. 6C, the micromotors (MnO_2_ and MnO_2_/Lac) were able to degrade almost 100% of urea, while blank experiments in the presence of only H_2_O_2_ show a minimal influence on urea removal. More importantly, as shown in Fig. 6D, the modified micromotors (MnO_2_/Lac) led to a higher generation of ammonia (31 ppm) in comparison with the blank (only H_2_O_2_) and bare MnO_2_ micromotors that generated only 1.9 and 2.5 ppm of ammonia, respectively.

To get an insight into the reaction mechanisms, we also determined the generation of nitrite and nitrate ions from urea degradation. As can be seen from Fig. S6,† the highest generation of nitrate ions was achieved by the MnO_2_/Lac micromotors, while nitrite ions were only detected for the experiments performed in the presence of bare MnO_2_ micromotors and the blank.

According to previous works, urea degradation is mainly mediated by the ˙OH radicals, which involves the conversion of amino groups from the urea into a nitro group, and finally carbamic acid. These carbamic acid can then undergo two oxidation pathways; (i) decomposition into HCO_3_^−^ and NH_4_^+^ by the reaction between ˙OH and e^−^ and (ii) decomposition into CO_2_ and NO_3_^−^ by only ˙OH radicals, leading to the formation of hydroxylamine and NO_2_^−^ ions.^2,8,51^ Therefore, the higher generation of ammonia by MnO_2_/Lac micromotors can be explained by considering that laccase generates one electron from the reduction of Cu(ii) to Cu(i), leading to the degradation of the resulting carbamic acid (*via* I pathway). Moreover, the lack of nitrite ions in the presence of MnO_2_/Lac micromotors also corroborate this result.

Additionally, we evaluated the generation of hydroxyl radicals by MnO_2_ and MnO_2_/Lac micromotors, which play an important role on the degradation of organic pollutants.^31,49^ For this purpose, we used terephthalic acid as a probe molecule to detect the generation of such radicals by photoluminescence. The hydroxylation of terephthalate produces its monohydroxylate isomer, which is a fluorescent molecule excited at a wavelength of 312 nm.^44^ In Fig. 6E, an increase in the fluorescence intensity of the isomer can be observed as the reaction proceeds in the presence of MnO_2_/Lac micromotors. This indicates that the generation of hydroxyl radicals increases over time. Moreover, a comparison of the performance by MnO_2_ and MnO_2_/Lac micromotors evidenced a higher generation of hydroxyl radicals by the latter (Fig. S7†). The generation of ˙OH radicals was also confirmed by electron paramagnetic resonance (EPR) (Fig. 6F). The spin adduct DMPO/˙OH profile was easily identified in all the measurements, observing the height ratio of 1 : 2 : 2 : 1 described in the literature.^45^ The largest signal was from the MnO_2_/Lac micromotors, while the peroxide showed a lower signal. The generation of such radicals by bare MnO_2_ micromotors was also corroborated by EPR measurements (Fig. S8†), where six peaks with the same height were identified.

As a precautionary step to enhance their environmental friendliness, the MnO_2_/Lac micromotors can be further modified with Fe_3_O_4_ nanoparticles. This modification allows for the micromotors to be easily recovered using external magnetic fields, ensuring their proper recovery and/or disposal, effectively minimizing any potential environmental impact.

## Conclusions

In this work, we successfully developed hybrid bubble-propelled tubular micromotors by combining sol–gel and hydrothermal reactions, utilizing silica as the scaffold and coating it with a MnO_2_ layer. To enhance their catalytic efficiency in degrading organic pollutants, such as RB and urea, we further functionalized the surface of the micromotors with laccase. Remarkably, we observed exceptional performance after enzyme loading, achieving almost 100% removal of RB. Additionally, we demonstrated the capabilities of such hybrid micromotors to generate ammonia from a urine-based pollutant, highlighting their potential for sustainable energy production. In parallel to these advancements, the utilization of a machine learning algorithm for the tracking of bubble-propelled micromotors was also documented in this research. This approach provides valuable insights into the motion and behaviour of the micromotors, facilitating their precise control and monitoring. Overall, this study represents an alternative approach towards the development of biocatalytic micromotors as a promising platform for the generation of green energy fuels.

## Author contributions

Conceptualization, K. V.; methodology, R. F., H. B., G. V. and K. V.; investigation, R. F. (synthesis and characterization of the micromotors) and H. B. (tracking experiments); writing – original draft, R. F., and H. B.; writing – review & editing, G. V., and K. V.; funding acquisition, G. V. and K. V.; resources, G. V. and K. V.; supervision, G. V. and K. V.

## Conflicts of interest

There are no conflicts to declare.

## Supplementary Material

NR-015-D3NR03804A-s001

NR-015-D3NR03804A-s002

NR-015-D3NR03804A-s003
