# Supplementary material for: Bubble-propelled micromotors for ammonia generation
Source: Nanoscale. 2023 Sep 18;15(38):15785–93. doi: 10.1039/d3nr03804a (PMC10551873; doi:10.1039/d3nr03804a)
Supplement: NR-015-D3NR03804A-s001 [file NR-015-D3NR03804A-s001.pdf]

Electronic Supplementary Material (ESI) for Nanoscale.
This journal is © The Royal Society of Chemistry 2023

Supplementary Information of "Bubble-Propelled Micromotors for Ammonia Generation"

Rebeca Ferrer Campos [a], Harshith Bachimanchi [b], Giovanni Volpe * [b] and Katherine Villa *[a]

a. Institute of Chemical Research of Catalonia (ICIQ), Av. Països Catalans, 16, Tarragona E-43007, Spain. Email: kvilla@iciq.es

b. Department of Physics, University of Gothenburg, Origovägen 6B, Gothenburg 41296, Sweden. Email: giovanni.volpe@physics.gu.se

This supplementary information file contains:

Supplementary Figures S1-S8

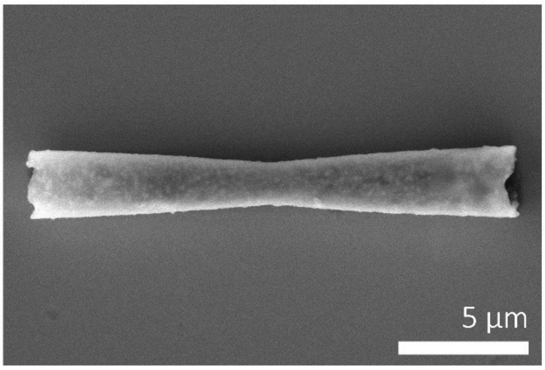

Figure S1. High-resolution FESEM image of a silica microtube.

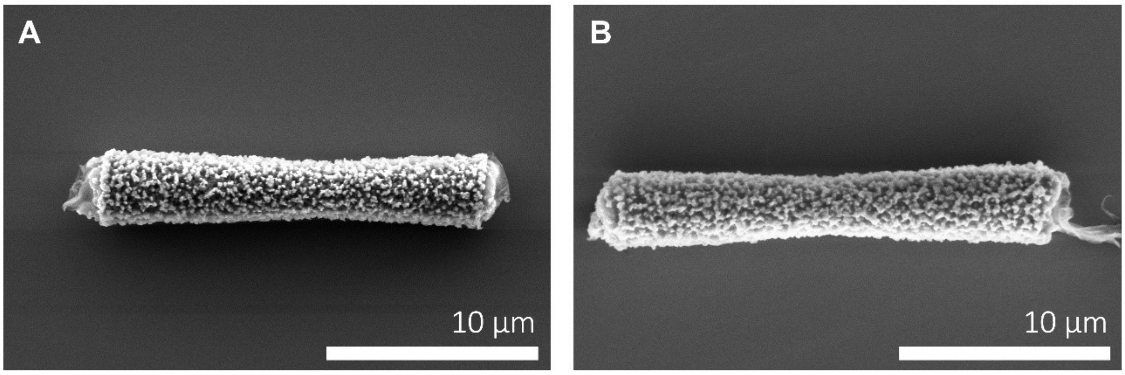

Figure S2. High-resolution FESEM images of a MnO_2 micromotor **A)** before and **B)** after the laccase functionalization.

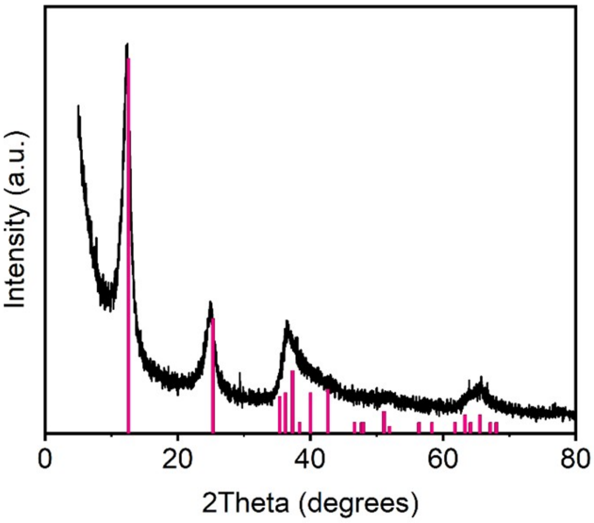

Figure S3. XRD of MnO_2 micromotors.

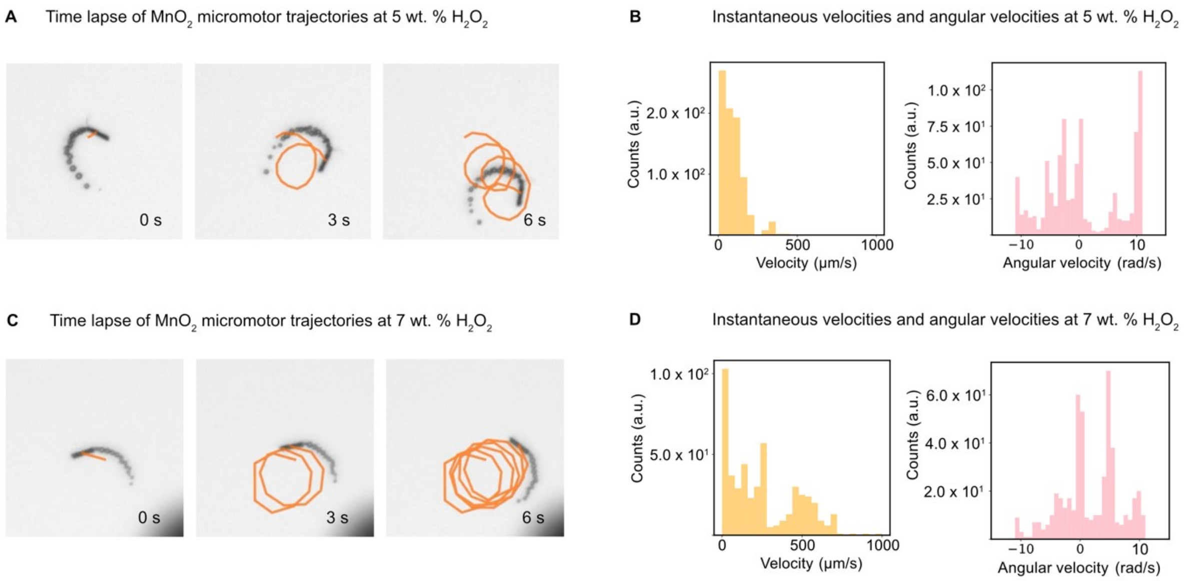

Figure S4. Motion characterization of the MnO_2 micromotors at different H_2O_2 concentrations. **A)** MnO_2 micromotors trajectories with 5% (w/w) H_2O_2. **B)** Velocities and angular velocities for the 5% (w/w) H_2O_2 collection of trajectories. **C)** MnO_2 micromotors trajectories with 7% (w/w) H_2O_2. **D)** Velocities and angular velocities for the 7% (w/w) H_2O_2 collection of trajectories.

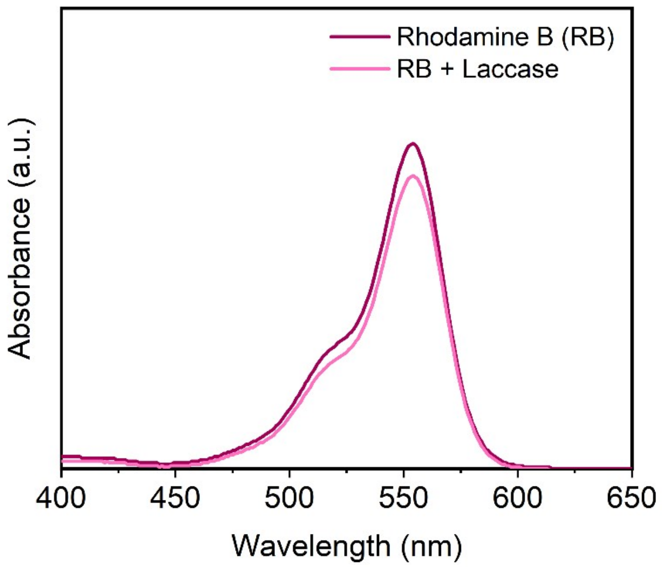

Figure S5. UV-vis spectra of RB with free laccase after 3h of reaction.

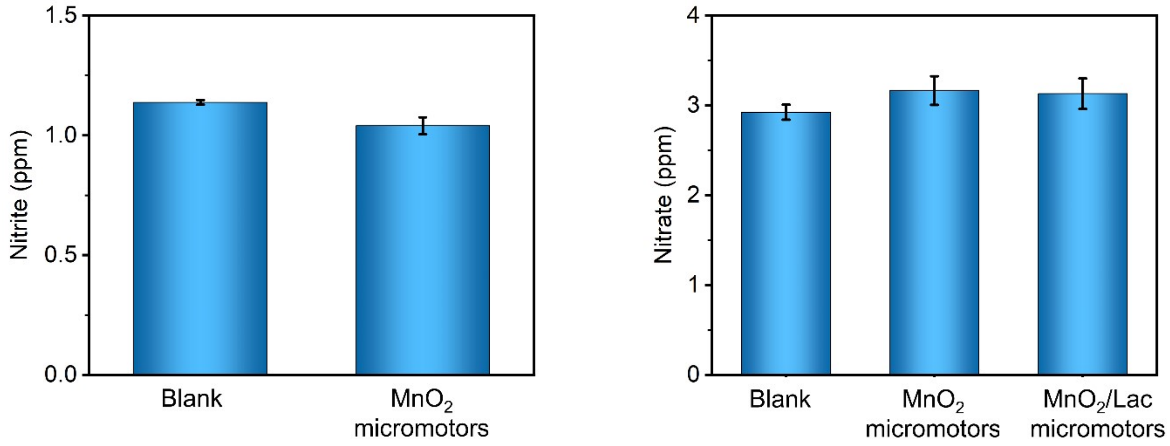

Figure S6. Ion chromatography results for the concentrations of **A)** nitrite and **B)** nitrate ions.

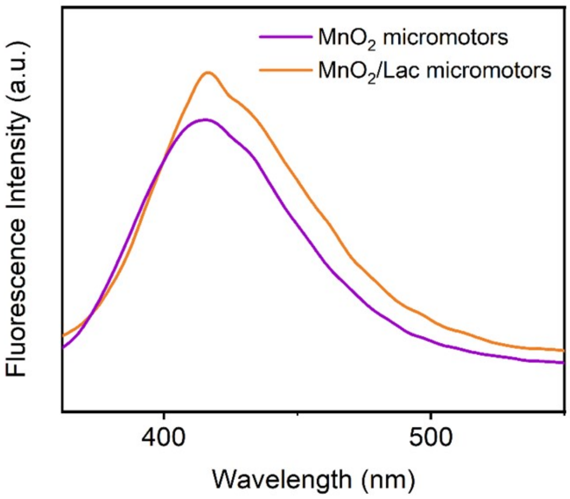

Figure S7. Fluorescence spectra of the terephthalate monohydroxylate isomer generated with MnO_2 and MnO_2/Lac micromotors at 15 min reaction time.

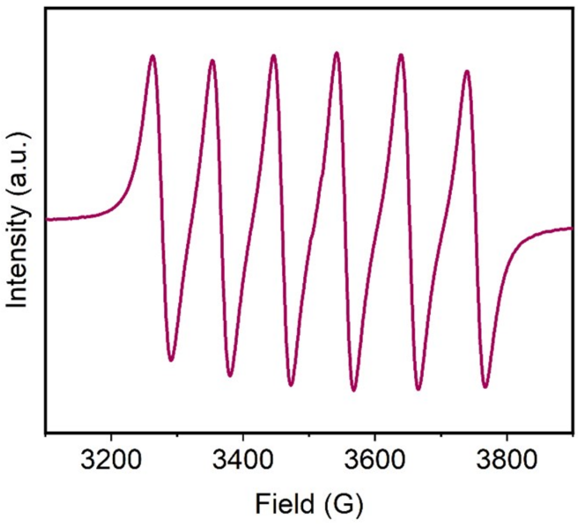

Figure S8. EPR spectrum of the MnO_2 micromotors.
